# Immune-related toxic epidermal necrolysis affecting trachea mucosal epithelium: a case report and literature review

**DOI:** 10.3389/fphar.2024.1454015

**Published:** 2024-10-18

**Authors:** Mingbo Zhang, Yang Fu, Yuxiao Song, Xia Gao, Jun Wang, Bicheng Zhang

**Affiliations:** ^1^ Cancer Center, Renmin Hospital of Wuhan University, Wuhan, China; ^2^ Department of Oncology and Hematology, Xiangyang Hospital, Hubei University of Chinese Medicine, Xiangyang, China; ^3^ Department of Oncology, The First Affiliated Hospital of Shandong First Medical University, Jinan, China

**Keywords:** PD-1, PD-L1, immunotherapy, immune-related adverse events, toxic epidermal necrolysis, fiberoptic bronchoscopy

## Abstract

**Background:**

Monoclonal antibodies against programmed cell death protein-1 (PD-1)/programmed death-ligand-1 (PD-L1) have emerged as critical tools in cancer treatment. However, concerns regarding their potential cutaneous and mucosal toxicity, along with severe complications, have drawn clinical attention. Further research is warranted to investigate the adverse reactions and treatment strategies associated with PD-1 monoclonal antibodies.

**Methods:**

We present a detailed case report of a laryngeal cancer patient who developed toxic epidermal necrolysis (TEN) after treatment with PD-1 monoclonal antibody. We analyzed the etiology, diagnosis, and treatment approaches by integrating clinical manifestations, pathological examinations, and literature research.

**Results:**

After PD-1 monoclonal antibody therapy, the patient exhibited systemic rash, bullae, and epidermal detachment, which subsequently involved the tracheal and bronchial mucosa, resulting in dyspnea. The patient recovered after treatments with steroids, macrolides, immunoglobulins, and etanercept, along with repeated removal of scabs via bronchoscopy. Literature reviewing suggests a potential association between PD-1 monoclonal antibodies and the pathogenesis of Steven Johnson’s Syndrome (SJS) and Toxic epidermal necrolysis (TEN), possibly due to immune dysregulation. Treatment consists of immediate discontinuation of suspicious drugs, essential supportive therapy, and systemic corticosteroid administration, with the addition of immunosuppressants and/or immunoglobulins needed.

**Conclusion:**

The mucocutaneous toxicity induced by PD-1 monoclonal antibodies is not limited to the surface of the skin but also in deep mucosal layers, potentially leading to life-threatening complications. Therefore, when using PD-1 monoclonal antibodies, clinicians should closely monitor adverse events and apply appropriate treatments as soon as possible to prevent severe complications.

## 1 Introduction

Immunotherapy has been utilized for various kinds of cancer, greatly transforming the panorama of malignant tumor treatment. With the growing use of drugs for immune therapy represented by immune checkpoint inhibitors (ICIs), immune-related adverse effects (irAEs) are drawing more and more attention. IrAEs range from mild diarrhea, fatigue, rash, to more severe events as immune-related pneumonia and myocarditis (Rached et al., 2024; Wang et al., 2019). Dermal toxicity is the most commonly seen, with its severity depending on clinical presentation and body surface area (BSA) affected classification (Collins et al., 2017): Grade 1 reaction include pathological erosions or erythema with no associated symptoms; Grade 2 reactions include Grade 1 reactions covering less than 50% of BSA except itching or other associated symptoms; Grade 3 reaction include symptomatic generalized erythema or rash, papules or blistering erosions or desquamations covering >50% of BSA; Grade 4 reaction include generalized exfoliative dermatitis or ulcerative dermatitis. Most skin adverse reactions were classified a grade 1 or grade 2. Severe (grade ≥3) events were less observed, such as Stevens-Johnson syndrome (SJS)/toxic epidermal necrolysis (TEN) with high mortality, which required more attention. This report discusses a case of TEN induced by programmed cell death-1 (PD-1) monoclonal antibody therapy, suggesting that TEN is not limited to body surface, but may also involve trachea and bronchial mucosa. However, its pathogenesis, clinical diagnosis and treatment strategy require further study.

## 2 Case presentation

A 60-year-old male patient visited our hospital with chief complaint of hoarseness accompanied by sensation of a foreign body in pharynxl in January 2023. A new organism was identified by laryngoscope. On 29 January 2023, the patient underwent a laryngoscopic biopsy and laryngectomy. Pathological examination revealed the presence of moderately differentiated, non-keratinized squamous cell carcinoma in both the supraglottic and glottic regions. Subsequently, on 2 February 2023, the patient underwent a tracheotomy, total laryngectomy with radical neck dissection, and tracheostomy reconstruction under general anesthesia. Postoperative pathology indicated: (a) an invasive squamous cell carcinoma of the larynx, keratinized type, with high-to-moderate differentiation, measuring 3.2 cm × 3 cm × 2 cm, exhibiting infiltration into the muscularis propria and multifocal nerve invasion, but no definitive intravascular tumor thrombus; (b) no malignancy detected in the incisal margins of the epiglottis and cricoid cartilage; (c) absence of cancer metastasis in the examined lymph nodes (0/55) in the right neck; and (d) immunohistochemical analysis demonstrated positive results for EGFR, Ki-67 (approximately 50% labeling index), scattered positivity for P53 (wild-type), and punctate positivity for P16. He was finally diagnosed as invasive squamous cell carcinoma of the larynx (pT2N0M0). On 6 April 2023, the patient commenced radiotherapy with a plan of 60Gy/30f targeting the cervical drainage area of CTV and the tumor bed post-surgery.

## 3 Diagnosis and treatment

**FIGURE 1:**
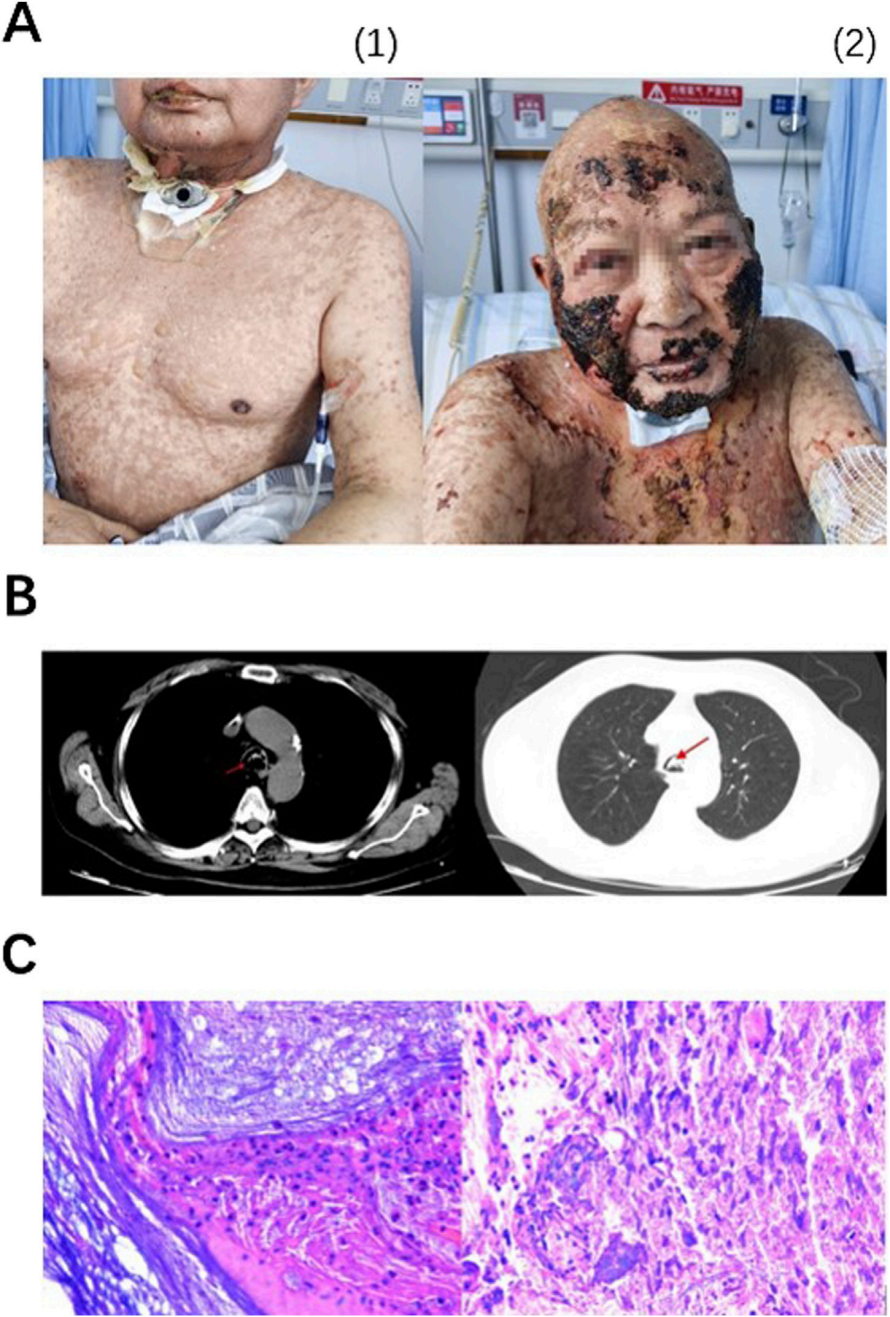
Lesions on skin and lip continued progressing from 3 January 2024 **(A1)** to 29 January 2024 **(A2)**, appearing first as MPR, bulla and then scabs. On 10 January 2024, emergency CT showed suspicious sputum plugs in the trachea and bilateral bronchi (indicated by arrows), no signs of interstitial pneumonia sighted **(B)**. Pathological examination (× 200) of foreign bodies in the airway revealed extensive infiltration of neutrophils, as well as a small amount of well differentiated, hyperkeratotic, and poorly keratinized squamous epithelial cells. Immunohistochemical results showed Ki-67 (+, low proliferation), P40 (−), and P53 (−) **(C)**.

On 11 December 2023, a MRI of nasopharynx and neck was performed due to obvious aggravation of submental and facial edema. MRI revealed new soft tissue mass located on the right side of tongue and the floor of the mouth, raising concerns about tumor recurrence. On 13 December 2023, the first cycle of chemotherapy combined with immunotherapy was performed as below: albumin paclitaxel 400 mg d1, carboplatin 480 mg d1, toripalimab (anti-PD-1 antibody) 240 mg d1, injected every 3 weeks. The biopsy indicated positive outcome of EGFR, on 27 December 2023, the patient received cetuximab at a dose of 700 mg. The next day, the patient developed rash with itching on the face and neck and blistering under the chin, which progressed to generalized rash with bullae after anti-allergic treatment with methylprednisolone 1 mg/kg per day (Figure 1A).

Due to the patient’s skin lesions, blisters BSA score exceeding 30%, disturbances in water and electrolyte balance. These factors, combined with the patient’s anti-tumor treatment regimen and poor response of anti-inflammatory and anti-allergic treatment, led to the diagnosis of immune-related TEN. On 31 December 2023, new treatment protocol for TEN was initiated. Methylprednisolone 2 mg/kg per day, mycophenolate mofetil capsules 1 g twice per day, intravenous immunoglobulin (IVIG) 20 g per day for 5 days and anti-tumor necrosis factor (TNF) therapy with etanercept 25 mg twice per week were given. The patient’s skin lesions gradually recovered.

On the evening of 10 January 2024, the patient reported experiencing dyspnea. The nurse aspirated sputum but no sputum was found. An emergency chest CT showed a potential sputum embolism in trachea and bilateral bronchioles (Figure 1B). On 11 January 2024, fiberoptic bronchoscopy was performed. Mucosa exfoliation, congestion and edema were observed under fiberoptic bronchoscopy. A large number of long sputum and blood crusts were seen in the main airway and right main bronchus, and the left main bronchus was completely blocked. After the doctor washed the trachea with normal saline, most of the sputum and blood crusts were successfully removed, and the patient’s dyspnea was significantly relieved after operation. The cleared tissues were sent for pathological examination, demonstrating that plenty of neutrophils and a number of T lymphocytes infiltrating and a few squamous epithelial cells of good differentiation. Hyperkeratosis and parakeratosis were observed in airway foreign bodies. Immunohistochemical results showed Ki-67 (+, low proliferation), P40 (−) and P53 (−) (Figure 1C). After that, the scab was removed under bronchoscope for many times. On 10 February 2024, the patient was principally relieved and discharged. The patient experienced G4 irAEs following the administration of immunotherapy in conjunction with chemotherapy. Subsequently, immunotherapy was discontinued, and the patient underwent four cycles of monotherapy utilizing albumin-conjugated paclitaxel in combination with platinum-based agents. Currently, the tumor is under effective control.

## 4 Discussion

### 4.1 Diagnosis and treatment of immune-related skin adverse reactions

ICIs are commonly used immunotherapeutic agents in clinical practice. Currently, the most widely used ICIs target PD-1/programmed death-ligand-1 (PD-L1) pathway. The status of cytotoxic T-lymphocyte-associated protein 4 (CTLA-4) inhibitors appears to be declining, while lymphocyte activation gene-3 (LAG-3) inhibitors are progressively gaining prominence. PD-1/PD-L1 pathway inhibits T cell activation, induces lymphocyte apoptosis and sustains autoimmune tolerance. In the tumor microenvironment, tumor cells with PD-L1 overexpression can interact with PD-1 on the surface of lymphocytes, surpressing lymphocyte function and thus evading immune surveillance and destruction. IrAEs in skin and mucosa have been documented to be associated with tumor immunotherapy, among which anti-PD-1 treatment more likely to induce skin-related reactions (Godfrey et al., 2024). Based on the patient’s clinical manifestations, signs, and immunotherapy history, we believe that the G4 mucocutaneous toxicity in this patient is related to the administration of a PD-1 monoclonal antibody.

Adverse events affecting the somatic skin after treatment with ICIs, if clearly drug-related, are collectively categorized as immune-related skin adverse reactions. These reactions encompass a range of conditions including rash, itching, blisters, psoriasis, lichenoid dermatitis, vitiligo, and the most severe forms of epidermal necrosis and exfoliation, including TEN, as illustrated in this case (Chen et al., 2024). Immune-related skin reactions usually occur early in treatment, such as days to weeks after administration, however, instances have also been documented following the cessation of immunotherapy. According to statistics, the median onset time of SJS/TEN is 24 days (Lu et al., 2023). Skin related adverse reactions are the most prevalent adverse reactions of ICIs (Basketter and Kimber, 2018). In addition, irAEs induced by ICIs may involve skin, gastrointestinal tract, liver, lungs, endocrine organs, etc. (Committee of Neoplastic Supportive-Care CA-CA and Cancer Clinical Chemotherapy Committee CA-CA, 2022). Conversely, adverse reactions such as vasculitis (Lee et al., 2024) and systemic sclerosis (Li and Xiong, 2024) are relatively infrequent.

A recent study utilizing the FDA Adverse Reaction Reporting System (Satoh et al., 2024) revealed that PD-1 inhibitors represent the predominant immunotherapies associated with the induction of SJS/TEN induction.(58.9%), followed by PD-1 inhibitors combined with CTLA-4 inhibitors (11.6%). Compared to conventional anticancer drugs, PD-L1 inhibitors ICIs were associated with increased mortality due to immune-related severe cutaneous adverse reactions (SCARs), with SJS 28.5% vs. 24.5% and TEN 55.3% vs. 46%. The association between SCARs and ICIs was independent of tumor status. The latest data show a general decline in the incidence of fatal irAEs after 2020, with the exception of muscle weakness and serious skin adverse reactions (Gougis et al., 2024). The occurrence of irAEs often indicates a good response to immunotherapy. However, its relationship to prognosis remains unclear. For now, the association between the occurrence of vitiligo and better prognosis is only identified in patients with melanoma (Cybulska-Stopa et al., 2022). In the context of other solid tumors, no reliable evidence linking cutaneous adverse reactions and the efficacy of ICIs has been found.

### 4.2 Immune-related TEN involving the trachea is rare

This case suggests that immune related TEN may not be limited to the epidermis but can also affect the tracheal mucosa, leading to inflammatory edema, necrosis, and even detachment of the mucosa, which may block the airway and ultimately lead to dyspnea.

IrAEs that lead to respiratory symptoms are usually immune related pneumonia rather than skin mucosal reactions. Common clinical manifestations of immune related pneumonia include dyspnea, coughing, fever, and hypoxia, which can also be asymptomatic. Radiological findings often reveal obvious lung changes such as acute interstitial pneumonia, cryptogenic tissue pneumonia, hypersensitive pneumonia, or non-specific interstitial pneumonia (Castanon, 2016). Compared to infectious pneumonia, fever caused by immune related pneumonia is relatively rare, but it is more prone to respiratory failure and may also be accompanied by other irAEs. Currently, there are no definitive biomarkers available to differentiate between infectious pneumonia from immune pneumonia, and both of them can exist simultaneously on the same patient (Infectiology Group et al., 2021). In addition, a considerable portion of respiratory distress caused by immunotherapy reported in recent years has also been linked to cardiotoxicity (Tajmir-Riahi et al., 2018).

Mechanical dyspnea resulting from immune-related skin toxicity involving trachea is rare. To date there barely exists report of immune-related SJS/TEN involving trachea and bronchial mucosa. CT examination of this patient after sudden dyspnea showed tracheal and bronchial obstruction. This obstruction was obviously alleviated after tracheal lavarage and blood scab removal via fiberoptic bronchoscopy. These findings suggest that it was airway obstruction rather than pneumonia that caused dyspnea. Histopathological analysis of the biopsy further indicated that the primary constiuant of obstruction was not sputum commonly seen, but a combination of bronchial mucosal fragments, bronchial secretions and blood clots. This suggests that the underlying pathology was acute inflammation involving tracheal mucosa resulting in mucosal hemorrhage, necrosis, exfoliation. This is distinct from the usual respiratory distress caused by direct lung damage from immune-mediated pneumonia.

### 4.3 Pathogenesis of drug-related SJS/TEN

SJS and TEN are classified as immune-mediated type IV hypersensitivity reactions, predominantly involving CD8^+^ T lymphocytes. CD8^+^ T cells have been identified as important mediators of blister formation (Chuenwipasakul et al., 2023). Earlier observations of TEN patients revealed a pan-T cell decrease at the focal site, characterized by infiltration of CD8^+^ T cells and a decrease in CD4^+^ T cells (Correia et al., 1993; Roujeau et al., 1985). There is also a negative correlation between dermal infiltration of CD8^+^ T cells and severity of acute ocular complications, alongside a positive correlation with serum interferon-γ (IFN-γ) levels (Chuenwipasakul et al., 2023). A seperate meta-analysis identified a distinct light distribution of rash in SJS/TEN patients and suggested that this may be related to photoactive drugs and UV exposure (McKinley et al., 2023). It has been posited that aberrant drug metabolism in patients (such as failure to remove active metabolites) induces T cell-mediated cytotoxic responses to drug antigens in keratinocytes, such as allopurinol (ALP) and its metabolite oxypurinol (OXP) (Mifsud et al., 2023). CD4^+^ T cells, like CD8^+^ T cells, secrete granolysin in SJS/TEN induced by carbamazepine (CBZ) under drug stimulation to mediate disease development (Jaruthamsophon et al., 2023).

Certain chemotherapy agents have been implicated in the induction of SJS/TEN symptoms themselves. For instance, methotrexate is known to cause toxic skin ulcers, or may manifest as severe phenotypes similar to SJS/TEN (Wang et al., 2023). The combination of carboplatin and Paclitaxel has also been reported to induce SJS in 2 cases (Gokulanathan et al., 2023). Findings of chemotherapy-induced skin adverse reactions local biopsy results can be similar to those described above. These reactions are classified as drug eruptions resulting from pharmacological agents, frequently accompanied by eosinophilia and systemic manifestations, and are also referred to as drug-induced hypersensitivity syndrome (DIHS) (Hung et al., 2024). At present, mechanisms by which chemical agents induce skin toxicity through T cells include: (a) chemical reactive compounds (i.e., drugs or their metabolites) covalently bind to their own proteins to form complexes as new antigens (Basketter and Kimber, 2018); (b) Drugs bind non-covalently to immune receptor proteins such as major histocompatibility complex (MHC) or T cell receptor (TCR) to activate the response (Pichler, 2008); (c) drugs penetrate into the interior of human leukocyte antigen B (HLA-B) molecule to change its structural specificity and thus change the antigen type presented (Ostrov et al., 2012).

Clinical and histopathological features of SJS/TEN induced by ICIs are similar to SJS/TEN induced by other drugs (Watanabe and Yamaguchi, 2023). CD8^+^ T cells are recognized as significant contributors to immunotherapy-related skin adverse reactions. Notably, PD-1/PD-L1 antibodies are of lower risk of maculopapular rashes (MPR) compared to CTLA-4 antibodies (Bottlaender et al., 2020; Curry et al., 2017; Minkis et al., 2013). For PD-1/PD-L1 inhibitor therapy, cross-immunity of CD8^+^ T cells to common antigens in skin and tumor tissues may be one of the causes of SJS/TEN, such as BP180, an anchor filament component involved in dermal-epidermal junction (Berner et al., 2019). While PD-L1 expression is generally low in skin keratinocytes, CD8^+^ T cell infiltration and PD-L1 expression have also been reported in glandular and basal skin biopsies from focal lesions in patients experiencing TEN associated with tumor immunotherapy (Li et al., 2023a; Vivar et al., 2017). Furthermore, PD-L1 expression levels are responsively up-regulated after ICIs treatments (Chen et al., 2018). The involvement of PD-L1 in preserving the integrity of dermal-epidermal junction and in mitigating the progression of TEN has been substantiated through additional murine studies (Goldinger et al., 2016; Okiyama and Katz, 2014).

Furthermore, PD-1 inhibitors have been observed to elicit cutaneous reactions characterized by gene expression profiles that resemble those of SJS and TEN, yet they differ from the profiles associated with acute skin graft-versus-host disease or maculopapular rash. The former consistently demonstrates the expression of major inflammatory chemokine-related proteins, including CXCL9, CXCL10 and CXCL11, as well as cytotoxic mediators such as Pore-Forming Protein 1 (PRF1) and granzyme B (GZMB), alongside a upregulation of pro-apoptotic molecules FAS ligand (FASLG) (Okiyama and Katz, 2014). The chemokine CXCL family mediates neutrophil migration to specific sites (Capucetti et al., 2020). This observation aligns with the pathological findings reported in previous case studies involving sputum and blood scabs. In instances of fatal cases from immune-related pneumonia, an increase in CD16^+^ T cells, CD57^+^CD8^+^ T cells expressing immune checkpoints (TIGIT, LAG3, TIM-3, PD-1), FCRL5+ B cells, and CCR2+CCR5+CD14^+^ monocytes (Yanagihara et al., 2024). These findings imply that the molecular mechanisms of SJS/TEN differ from that of immune-associated pneumonia. At present, the potential mechanisms of SJS/TEN induced by immunotherapy include: (a) excessive activation of T lymphocytes; (b) increase of inflammatory factors (such as TNF-α); (c) exposure of cross antigens (Basketter and Kimber, 2018).

### 4.4 Treatment of immune related SJS/TEN

According to the guidelines from National Comprehensive Cancer Network (NCCN) and Chinese Society of Clinical Oncology, corticosteroids are the most mainstream treatment for irAEs. However, approximately 10% of cases remain hormone non-responsive (i.e., hormone-refractory irAEs) and irAEs cannot be reduced (i.e., hormone-dependent irAEs) (Ruf et al., 2024). Given that the pathophysiology of irAEs extends beyond T cell immunotoxicity, it is imperative to consider adjunctive therapies in conjunction with systemic hormone administration for cases that are severe, refractory, or involve multiple organ systems. Such therapies may include immunosuppressive combinations, such as mycophenolate mofetil, cyclosporine, various novel monoclonal antibodies, intravenous immunoglobulin (IVIG), and/or plasma exchange therapy (Saw et al., 2017). There is still a lack of robust evidence for the effects of nonsteroidal anti-inflammatory drugs. Studies have shown that the administration of aspirin can effectively reduce the incidence of rash and SJS in patients with ICIs. However, this benefit is accompanied by an increased risk of adverse effects, including anemia, colitis, myocarditis, pneumonia, and other related conditions (Yang et al., 2022).

Studies have shown that patients with bullous dermatitis exhibit elevated levels of TNF - α expression in their serum or vesicular fluid (Stewart et al., 2024), Consequently, anti-TNF therapies can be considered an option, such as Infliximab (Kachi et al., 2024), etanercept (Ornstein et al., 2019). Infliximab and etanercept are both commonly used in anti-TNF therapies, with some differences in their mechanisms of action. Infliximab targets TNF-α, while etanercept competitively inhibits the binding of both TNF-α and TNF-β to cell surface TNF receptors. The Hepatotoxicity of Infliximab makes it unsuitable for treatment of autoimmune hepatitis (Jacobsen et al., 2022). Though etanercept is prohibited in patients with severe infections, there is still reports on its efficacy in patients suffering from open pulmonary tuberculosis, septicemia and fungal sepsis (Peng et al., 2024). In this case, we chose etanercept as the anti-TNF-α agent, yielding favorable outcomes.

Through a comprehensive review of literature, we have compiled some SJS/TEN case reports related to ICIs published in the past 10 years (Table 1). Each case report emphasizes the necessity of immediate cessation of the implicated medications, indispensable supportive therapy, systemic use of glucocorticoids, and the addition of immunosuppressants and/or IVIG depending on conditions. Due to the potential high lethality of SJS/TEN, clinical doctors must closely observe the patient’s condition when using ICIs to identify potential adverse reactions as early as possible. Timely and appropriate medical interventions are crucial in managing these serious conditions.

**TABLE 1:** Case reports on ICI-induced TEN recent years.

	Publication year	Authors	Cancer type	Patient age/sex	Therapy	Treatment for TEN	Outcome	Biopsy of lesion area
1. Li et al. (2023b)	2023	Li X.	Lung adenocarcinoma	76/M	Sintilimab with paclitaxel liposome	Methylprednisolone and immunoglobulin, Mupirocin ointment applied to the infected area	Success	Infiltration of CD4 and CD8 positive T lymphocytes
2. Li et al. (2023a)	2023	Li X.	Non-small cell lung cancer (NSCLC) and prostate cancer	75/M	Anlotinib and pembrolizumab with androgen deprivation therapy	Corticosteroids, gamma globulin, and immunosuppressants	Success	Refused
3. Kumar et al. (2024)	2024	Kumar S.	Esophageal squamous cell carcinoma	46/M	Capecitabine and oxaliplatin (CAPEOX) combination chemotherapy	Intravenous steroids, oral antihistaminic, topical steroids, topical astringent, and antipruritic lotion along with supportive care	Success	Predominantly necrotic tissue with entrapped necrotic keratinocytes, inflammatory exudates
4. Logan et al. (2020)	2020	Logan	Melanoma	62/M	Ipilimumab and nivolumab	Immunoglobulin IV, G-CSF and cyclosporine	Died of systematic failure	Not mentioned
5. Yang et al. (2022)	2022	Yang H.	Thymic squamous cell carcinoma	82/M	Sintilimab	Methylprednisolone and immunoglobulin	Rash disappeared but died of pneumonia	Detachment of the epidermis and mucous membrane, subepidermal blisters. perivascular lymphocytes accumulation

## Data Availability

The raw data supporting the conclusions of this article will be made available by the authors, without undue reservation.

## Abbreviations

PD-1, Programmed cell death protein-1; PD-L1, Programmed death-ligand-1; TEN, Toxic epidermal necrolysis; ICIs, Immune checkpoint inhibitors; irAEs, Immune-related adverse effects; BSA, Body surface area; SJS, Stevens-Johnson syndrome; EGFR, Epidermal growth factor receptor; IVIG, Intravenous immunoglobulin; TNF, Tumor necrosis factor; CTLA-4, Cytotoxic T-lymphocyte-associated protein 4; LAG-3, Lymphocyte activation gene-3; SCARs, Severe cutaneous adverse reactions; IFN-γ, Interferon-γ; ALP, Allopurinol; OXP, Oxypurinol; CBZ, Carbamazepine; DIHS, Drug-induced hypersensitivity syndrome; MHC, Major histocompatibility complex; TCR, T cell receptor; HLA-B, Human leukocyte antigen B; MPR, Maculopapular rashes; CXCL, C-X-C motif chemokine ligand; PRF1, Pore-forming protein 1; GZMB, Granzyme B; FASLG, FAS ligand; TIGIT, T cell immunoreceptor with Ig and ITIM domains; TIM-3, T cell immunoglobulin-3; FCRL5, Fc receptor-like protein 5; CCR, C-C chemokine receptor.
